# Study of Occupational Safety Risks in Prefabricated Building Hoisting Construction Based on HFACS-PH and SEM

**DOI:** 10.3390/ijerph19031550

**Published:** 2022-01-29

**Authors:** Yinghui Song, Junwu Wang, Denghui Liu, Feng Guo

**Affiliations:** 1 School of Civil Engineering and Architecture, Wuhan University of Technology, Wuhan 430070, China; songyinghui@whut.edu.cn (Y.S.); 267544@whut.edu.cn (J.W.); guofeng777@whut.edu.cn (F.G.); 2 Sanya Science and Education Innovation Park, Wuhan University of Technology, Sanya 572025, China; 3 China Construction First Group Corporation Limited, Beijing 100161, China

**Keywords:** occupational safety, prefabricated building, hoisting construction phase, human factors analysis and classification system, structural equation modeling

## Abstract

As the concern for environmental pollution and occupational safety caused by the construction industry is gradually increasing worldwide, the prefabricated building model has become a type of construction promoted by sustainable societies. In China, the management codes of prefabricated buildings are not mature enough and safety accidents occur frequently during the construction process. Therefore, how to analyze and determine the main factors that affect the safety of the construction of prefabricated buildings has become a problem to protect the lives and health of construction workers. In this study, we focused our research on the accident-prone component-hoisting construction phase. First, through the questionnaire and accident data, the traditional human factors analysis and classification system (HFACS) was improved into the HFACS–prefabricated building hoisting (PH) risk model. This study also established a comprehensive safety prevention and control system for the component-hoisting process of prefabricated buildings by combining the factor analysis of using structural equation modeling (SEM). The prevention and control measures to avoid the occurrence of prefabricated building component-hoisting accidents were also proposed from four aspects: external environment, organizational factors, prerequisites for triggering accidents, and unsafe leadership behaviors. The results showed the following: (1) For the external environment, occupational safety and health system standards should be established and safety supervision responsibilities should be implemented. (2) For organizational factors, safety management systems should be improved with more capital investment. (3) For unsafe leadership behaviors, safety education and training should be strengthened to ensure workers’ optimal physical and psychological states. (4) For the prerequisite of accidents, it is necessary to create a good hoisting work environment.

## 1. Introduction

With the development of the global economy, the urbanization and industrialization of underdeveloped countries are deepening. In the process of advocating for the sustainable development of global resources [1], it is noted that the traditional construction industry is undoubtedly a heavy resource consumption and carbon-emission-intensive industry [2]. To that end, the Chinese Government had proposed a Fourteenth Five-Year Plan and a vision for 2035 [3], which would make great efforts to develop prefabricated housing and promote the sustainable development of the world’s environment. Meanwhile, a cornerstone of the construction industry is engineering safety and worker safety.

According to data published by the Ministry of Housing and Urban–Rural Development, 96.17% of safety accidents in construction projects between 2009 and 2020 were caused by human and organizational factors (HOFs). With the development of infrastructure projects in China, the number of accidents in construction projects has correspondingly increased [4]. In 2020, according to publicly available information alone, 284 safety incidents occurred in the construction of prefabricated buildings, almost two-thirds of which occurred during the component-hoisting phase, with an average of 1.63 work-related accidents per component-hoisting phase per building [5]. Therefore, it is of great significance to understand the types and influencing factors of prefabricated building construction accidents. It also ensures worker safety and reduces the overall casualty rate from safety accidents in the construction industry [6].

The biggest difference between prefabricated buildings and traditional buildings lies in the construction process. The construction of traditional buildings only needs the transport of raw materials through the crane tower to the construction floor for in situ pouring. Meanwhile, for prefabricated buildings, the prefabricated components are precasted in the factory and are transported and spliced through a suspended tower in the construction site. Therefore, the HOFs prevention and control systems applied to traditional buildings have great defects for prefabricated buildings [7], and there are many influencing factors that are not common to both types of buildings that need to be investigated and analyzed again [8].

At present, the management technology of prefabricated units in China is not mature enough [9]. Compared with developed countries, there are still many deficiencies in the regulation of the industry. With regard to the impact of accidents in prefabricated buildings, most accidents occur during the lifting and installation of components at construction sites, where the robustness of construction planning is extremely fragile. During construction, there are many reasons for lifting accidents, any one of which will threaten the safety of workers. In addition, workers’ behaviors and the relationship between workers and organizations can also affect workers’ occupational safety throughout construction projects.

To sum up, given the background of China’s prefabricated building industry, it has become an urgent matter to analyze the causes of the hoisting accidents of components, put forward a valid prevention and control system for the hoisting accidents of components, protect the safety and health of workers, and reduce the probability of accidents. Based on the investigation of construction accidents of prefabricated units in China from 2010 to 2020, this study established an evaluation system of hoisting safety factors of prefabricated units and improved the original HFACS to establish an HFACS-PH model via modifications with SEM. Then, the numerical calculation was simulated using AMOS 26, which proposed corresponding solutions to reduce the accident rate.

The remainder of the present paper is organized as follows. Section 2 summarizes the previous scholars’ investigations on the construction process of prefabricated building hoisting and the methods used; Section 3 introduces the model, the 241 questionnaires, and related social information; Section 4 details the model improvement and establishes a mathematical model suitable for the study; Section 5 presents the model validation and solution; and Section 6 discusses the analysis results from four aspects: external environment, organizational influence, unsafe leadership behaviors and prerequisites for accidents, and presents the prevention and control system. In addition, it summarizes the whole study and provides a theoretical basis for further research.

## 2. Literature Review

### 2.1. Risk Identification of Safety Accidents in Prefabricated Buildings

In recent years, with the development of the prefabricated building model in China, some theoretical research results were obtained in the field of prefabricated building safety risk identification. Wang counted and analyzed a large amount of literature to design a questionnaire on the construction risks of prefabricated buildings using the theory of planned behavior in terms of the obvious differences that exist between prefabricated buildings and traditional cast-in-site buildings, and summarized the relevant influencing factors that lead to safety accidents in prefabricated buildings. Finally, he also analyzed the differences between these influencing factors and those in traditional cast-in-place buildings [10]. Many other scholars [11,12,13,14,15] conducted behavioral perception studies to analyze risk factors from management perspectives. Suraji (2001) [16] considered both the short-range and long-range effects during construction and found that different risk factors can interact. Suo (2017) [17] considered the influence of external objects and started with the auxiliary equipment of workers to establish a safety risk material analysis model. Wu (2010) [18] used electronic vision technology to identify the workers’ behaviors before accidents and then collected the data through the wireless sensor network to assess risk behaviors. Mohamed (2002) [19] focused on external influences, such as climate, and established a model of the interaction between workers’ occupational behaviors and climate. Yi (2013) [20] conducted research on the work and rest patterns of workers. After investigating related accidents, it was found that workers’ schedules are also one of the causes of risks. In summary, this study expanded on the contributions made by previous scholars in this area, focusing on how objective factors affect workers’ occupational safety.

### 2.2. Safety Management in Hoisting Process

For the safety management research of the component hoisting construction phase, most literature focuses on the layout and machinery. Scholars, such as Lu (2021) and Adrian (2015) [21,22,23,24,25,26], started with the hoisting construction process, then managed and controlled safety accidents from the perspective of the facility layout and design planning of the construction site. Although such research can plan accident areas through algorithms, it still cannot reduce the probability of accidents at the source. Liu (2021) [27] studied the correlation of risk factors in the lifting process using digital twin technology, which improved the efficiency of parallel accident handling. Zhao (2021) [28] managed the prefabricated component (PC) hoisting process through IoT sensing technology and realized an accident warning function after constantly learning the occurrence of accidents. Arashpour (2015) [29] performed multi-objective optimization of processes to avoid crossover between different processes, thus preventing safety accidents in the case of multiple tower cranes. Ma (2018) [30] used building information modeling to make the construction process more coordinated and avoid mechanical collisions. Shin (2015) [31] analyzed the causes of accidents related to tower crane disassembly and installation from 2001 to 2011 [32] and conducted systematic research on the hoisting safety management system in terms of workers’ occupational specifications and mechanical performance and quality. At present, most researchers focus on layout planning and process management, and there is still a big gap in the research on the causes of accidents in the hoisting process.

### 2.3. Application of the HFACS and SEM in Safety Accident Analysis

The composition of the causes of safety accidents is always complicated. HFACS, as a multi-stage accident cause decomposition model, has been studied in many different fields. Xia (2018) [33] improved the traditional HFACS, using a model with 5 stages and 18 influencing factors to analyze human-caused accidents in construction projects. Xu (2021) [34] analyzed aviation accidents with an HFACS model and proposed a training system for air traffic controllers. Hsieh (2018) [35] used an HFACS to decompose the causes of human-related medical error and concluded that the relationship between people and organizations is the main factor leading to malpractice. Liu (2018) [36] analyzed accidents on the basis of the original HFACS model and expanded the correlation between factors [37] to improve it to the HFACS-CM model, establishing a prevention and control system to manage human-related accident factors. Because the causes of safety accidents come from different subjects, it is more suitable to use the SEM method when determining the sensitivity factors. Xie (2021) [38] analyzed the family social needs of construction workers through the SEM method to improve workers’ safety behaviors on construction sites from the perspective of family harmony. Liang (2021) [39] established a mediating effect between worker responsibility and safety accidents to study its effect on workers’ safety behavior centered on worker emotions. Fugas (2012) [40] combined the safety climate with planned behavior theories and used SEM to verify the correlation between organizational safety climate and active safety behavior. Gao (2016) [41] and Guo (2016) [42] used SEM to verify the relationship between unsafe leadership behaviors and organizational relationships and worker safety behaviors when exploring the relationship between multi-level safety climate and safety performance in construction safety accidents and put forward suggestions to reduce the probability of accidents. In the above-mentioned literature, scholars mostly focused on the influence of workers’ subjective factors on engineering safety accidents, ignoring the objective factors that are the prerequisite for accidents. Without the subjective influence of workers, these objective factors will also lead to engineering accidents. Therefore, this study will summarize the shortcomings of previous studies and improve the analysis of the existing research base.

## 3. Preparation and Model Framework

### 3.1. Accidents Data Investigation and Analysis

**Figure 1:**
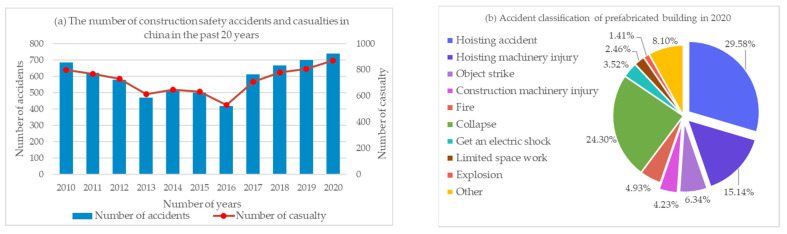
Annual engineering accidents statistical information: (**a**) the number of construction safety accidents and casualties in China in the past 11 years; (**b**) accident classification of prefabricated buildings in 2020.

The research foundation of this study was based on the detailed data from real and reliable engineering safety accidents. According to the statistics of safety accidents and casualties in construction projects in China in the past 11 years [4,43], the statistics of engineering accidents are shown in Figure 1.

According to the statistics on engineering safety accidents and casualties from 2010 to 2020 released by the Ministry of Housing and Urban–Rural Development of China and the statistics on the classification of safety accidents in prefabricated building projects in 2020, it is known that the average works injury rate per accident is more than 1 [4]. In safety accidents, hoisting accidents, hoisting machinery injuries, object strikes, and construction machinery injuries all occurred in the hoisting phase of components, accounting for 55% of the total number of accidents [44]. Therefore, this study analyzed the accidents investigation report and established the accident factors evaluation table.

### 3.2. Sample Analysis of Survey Object Data

The prefabrication project of Shuangyashan Chengxiang Construction and Installation Company is located in Shuangyashan City, Heilongjiang Province, covering an area of 216,943 square meters, with two floors underground and more than 15 floors above ground. This study was conducted by analyzing a large amount of literature and interviewing several qualification experts who have been working in the construction industry for many years. Based on the statistics of the interview results and combined with the classification data of major engineering safety accidents between 2010 and 2020 [4,5], an assessment table of accident factors for the hoisting of prefabricated building components was established. A questionnaire was set up on the basis of a five-level Likert [45] scale for the assessment of accident factors for the hoisting of prefabricated building components, and relevant construction units, practitioners, and experts were invited to fill in the questionnaire. The factors for accidents of prefabricated building components hoisting table are shown in Table 1.

**Figure 2:**
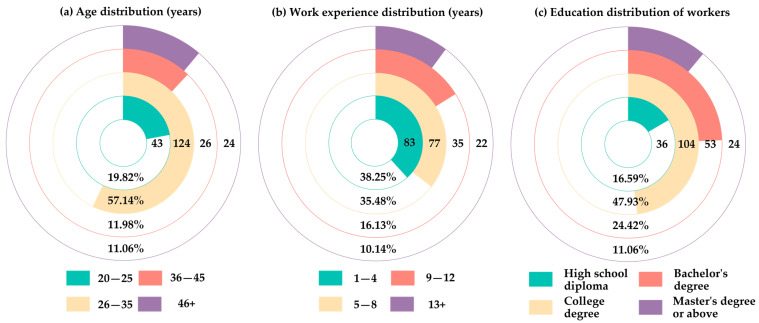
The age distribution, employment experience, and educational background of the participants.

In the above table, a score of 5 represents “highest impact”, 4 “high impact”, 3 “moderate impact”, 2 “slight impact”, and 1 “almost no impact”. A total of 241 questionnaires were distributed, and a total of 217 valid questionnaires were given back, with an effective rate of 90.04%. The age distribution, employment experience, and educational background of the participants are shown in Figure 2.

All the questionnaire respondents were trained in prefabricated building construction specifications and were members of the assembly building project under construction. Furthermore, among the respondents of the 217 valid questionnaires, there were 22 experts with senior engineer titles, 57 technicians with intermediate engineer titles, 23 construction site supervisors, 19 engineering equipment and facility operators, and 115 construction workers.

### 3.3. HFACS Model Theory

**Figure 3:**
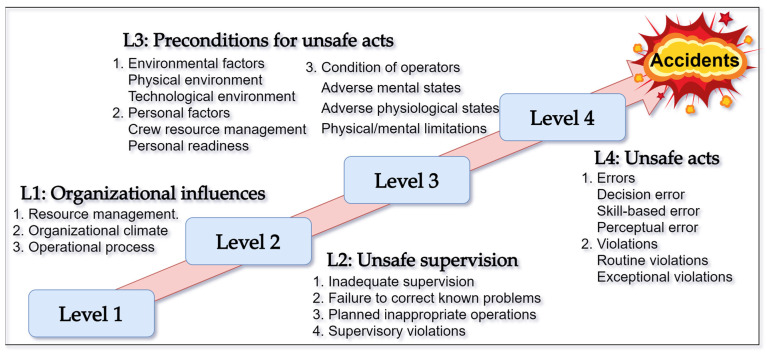
Original model diagram of the HFACS.

There are many applicable models for HOFs. By summarizing the research of previous scholars, it was found that the HFACS is the most consistent with the research purpose of this research. The HFACS model divides the factors of safety accidents into four major categories: organizational influence, unsafe leadership behaviors, prerequisites for causing accidents, and unsafe behaviors, as well as 12 sub-categories distributed amongst the major categories. The model diagram is shown in Figure 3.

The model mainly considers the relationships between the four major categories, the absence of any one of which may cause a safety incident, and the original model ignores the impact of group effects on the outside of the organization, as well as the lack of unsafe leadership behaviors on factors. Therefore, the original model needs to be improved regarding the analysis of the cause of the prefabricated building hoisting accidents.

### 3.4. SEM Theory

Structural equation modeling (SEM), an important method for processing and analyzing multi-dimensional arrays, is essentially a statistical method for covariance matrix analysis of the relationship between variables. It has a wide application in economics, management, social psychology, and engineering. When the evaluation index system is established in these different subject areas, there is a description method, called LA (latent variable), that no indicator can comprehensively and intuitively describe and accurately measure the occurrence of factors such as the development prospects of a company, the partnership with other companies, and the social tolerance of the company. It is necessary to quantitatively analyze these LAs with a numerical conversion method of OIs (observable indicators) and to convert them into values that can be expressed in numbers. OIs can estimate predictive models that contain latent variables, complex independent variables, and dependent variables by correlating the factors in each evaluation system. Similar to the traditional linear regression analysis prediction model, errors in the dependent variable are allowable, but it is necessary to ensure that the independent variable is accurate [46], and an ideal situation can be assumed to avoid dependent variable error from having a severe impact on the entire predictive model.

SEM can be divided into two stages. The first stage is the measurement model, which is used to specify the relationship between factors in the index system and the LA. The second one is a structural model that is standardized to reflect the correlation between the LA and the OIs [47]. In the measurement model, independent variable X and dependent variables Y are defined by introducing OIs. The logical relationship between the two variables can be represented by the measurement matrix. The calculation methods of independent variables and dependent variables are shown in Formulas (1) and (2).

(1) $$X=\Gamma_{X}\gamma +\varepsilon$$

(2) $$Y=\Gamma_{Y}\delta +\zeta$$

In the structural model, there is a mathematical calculation relationship between the independent variable and dependent variables, as shown in Formula (3).

(3) η=αγ+βδ+θ

**Figure 4:**
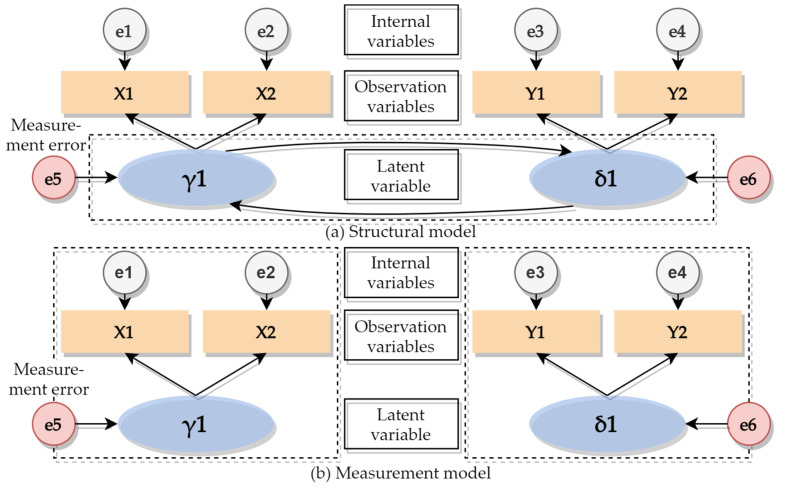
Construction diagram of the measurement model and structural model.

In SEM, the observed variables are available as direct numerical values, while the LA is not [48]. The composition diagram of the measurement and the structural models are shown in Figure 4.

## 4. Model Establishment

### 4.1. Improved HFACS Model

Combined with the current situation of prefabricated building component-hoisting construction and the statistics of component-hoisting accident cases, this study proposed the HFACS-PH model as a way to improve the original HFACS model. First, this study added external influences and considered their impacts on the whole organization. Second, this research analyzed the impact of unsafe leadership behaviors on objective factors and environmental factors on the construction site. Finally, by analyzing the accidents counted, this study obtained the factors that can trigger component-hoisting accidents among the physical factors and construction site environmental factors. Since the traditional HFACS causative model has been widely accepted in many fields, such as mines, plants, and oceans, it is reasonable to make relevant improvements in this study.

#### 4.1.1. External Environment

The external environmental factors include economic, policy, industry management factors, and historical factors. From the perspective of economic factors, the development process of prefabricated buildings is mainly affected by three stakeholders: the government, developers, and consumers. Construction companies and developers are communities of interest, and more and more companies are willing to study the construction of prefabricated buildings. Promoting the economical development of prefabricated buildings will enable construction companies in China to further understand the construction process of the buildings, thereby reducing the occurrence of safety accidents.

In terms of policy factors, the development of prefabricated buildings in China has entered a new stage. Since prefabricated buildings were vigorously promoted in 2016, China has built a total of 630 million square meters of new prefabricated buildings in 2020 [49], which is a 50% increase compared with the previous 19 years [50]. The related industry chain is also developing rapidly. These developments have made prefabricated construction industries more complete in China.

In terms of industry management factors, China’s construction industry is currently managed in a standardized manner, but a statistical analysis of accidents over the past 11 years shows that poor practices of relevant regulatory authorities are the main causes of safety accidents. If the relevant supervisory departments fail to perform their duties properly, it will lead to safety accidents; when workers work without safety, the construction enterprises will suffer serious losses, which will bring bad influence to the whole industry.

From historical factors, traditional construction methods have matured in China, and construction companies are not willing to change them. In addition, multi-party coordination factors should be considered during the hoisting and construction of prefabricated buildings. Some construction units still follow the traditional hoisting construction method, which makes the hidden factors that may cause accidents not obvious, gradually increasing the potential safety risks.

#### 4.1.2. Prerequisites of Accidents

According to the traditional accident causation theory [51], the analysis of safety accidents often starts from the personnel, external objects, environment, and management. In the original HFACS model, the prerequisites for the accidents are the personnel, environment, and management. From the perspective of the original model theory analysis, on the one hand, the model claims that unsafe leadership behaviors will not lead to changes in external factors; on the other hand, it considers that external factors will not lead to unsafe behaviors, which is reasonable. However, in the construction process of prefabricated buildings, external factors will be affected by organizational factors [52]. For the hoisting accidents of prefabricated building components, the unsafe leadership behaviors have an indirect influence on workers’ operation, thus causing hoisting accidents [53].

#### 4.1.3. HFACS-PH Model

**Figure 5:**
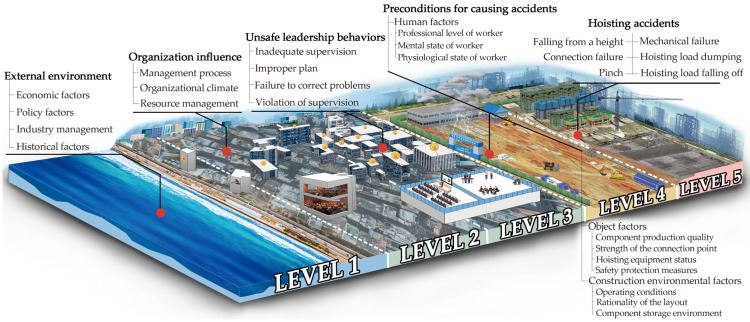
HFACS-PH model diagram.

The relevant influencing factors were modified and simplified through the above statistical analysis of safety accident investigations in China over the past 11 years and interviews with a large number of relevant experts. The organizational influencing factors in the traditional HFACS model were retained, and then external causal factors were added to the prerequisites for the accidents. The traditional HFACS model does not consider the influence of the external environment on the safety accidents in the whole project, and the preconditions and the classifications of unsafe behaviors in the original model are not suitable for assembly building projects. Therefore, in this study, the traditional HFACS model was improved and applied by combining the risk-causing factors of prefabricated buildings summarized in the previous paper [35] by adding external environmental influences and changing “Unsafe supervision, Preconceptions for unsafe acts and Unsafe acts” in the original model to “Unsafe leadership behaviors, Preconditions for unsafe acts and Unsafe leadership behaviors” to establish the HFACS-PH model for the analysis of the causes of safety accidents in the hoisting construction phase of assembly building projects. The HFACS-PH model diagram is shown in Figure 5 below.

### 4.2. SEM with Mediation Effect

The SEM method mainly analyzes the correlation between variables through the covariance matrix of the variables. By establishing a causal model and making a path analysis diagram, the parameters can be analyzed via covariance, factor, and path analyses. Based on the accident statistics of prefabricated buildings and the survey of experts’ opinions in the above study [38,40], the relationship between unsafe personnel behaviors and their effects was established by utilizing the HFACS-PH model, and the model was built and visually expressed by using AMOS 26 software. The relationship between unsafe behavior and its influencing factors can be found through an engineering accidents investigation and literature research. We proposed the following hypotheses:

**Hypothesis** **1** **(H1)**. *The external environment has an impact on prefabricated-component-hoisting accidents*.

**Hypothesis** **2** **(H2)**. *Organizational factors have an impact on prefabricated-component-hoisting accidents*.

**Hypothesis** **3** **(H3)**. *Unsafe leadership has an impact on prefabricated-component-hoisting accidents*.

**Hypothesis** **4** **(H4)**. *The preconditions that caused the accidents have an impact on precast-component-hoisting accidents*.

**Hypothesis** **5** **(H5)**. *The external environment has an impact on unsafe leadership behaviors*.

**Hypothesis** **6** **(H6)**. *The external environment has an impact on the prerequisites of accidents*.

**Hypothesis** **7** **(H7)**. *The external environment has a significant impact on organizational factors*.

**Hypothesis** **8** **(H8)**. *Organizational factors have an impact on unsafe leadership behaviors*.

**Hypothesis** **9** **(H9)**. *Organizational factors have an impact on the prerequisites of accidents*.

**Hypothesis** **10** **(H10)**. *Unsafe leadership behaviors have an impact on the prerequisites of accidents*.

**Figure 6:**
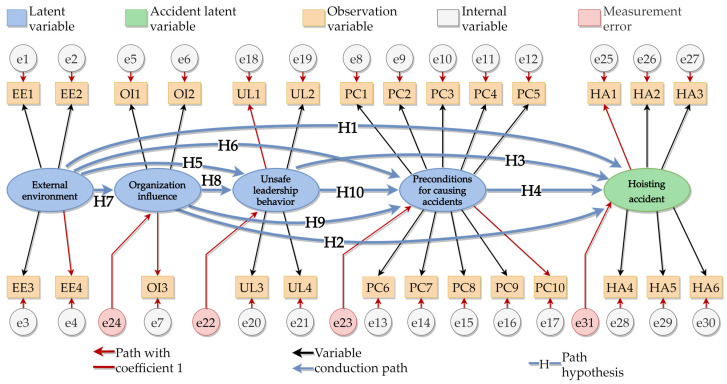
Model of factors affecting hoisting accidents of prefabricated building components.

According to the above hypotheses, the structural model of the factors affecting the hoisting accidents of prefabricated building components is shown in the following Figure 6.

## 5. Results

### 5.1. Case Study

This study analyzed and verified the 217 questionnaires collected from the prefabricated projects of Shuangyashan Chengxiang Construction and Installation Company and the statistical information of China’s prefabricated building construction safety accidents from 2010 to 2020.

#### 5.1.1. Combination Reliability and Correlation Analysis

The results of the questionnaires were tallied and the statistics were summarized in SPSS (IBM, 26, Armonk, NY, USA) to standardize the data. The data were processed with a combination of reliability and correlation analyses, and the processing relied on Formulas (4) and (5), as shown below.

(4) $$CR=\left(\sum \lambda^{2}\right)/\left(\left(\sum \lambda^{2}\right)+\sum \delta \right)$$

(5) $$AVE=\left(\sum \lambda^{2}\right)/n$$

In previous studies, scholars usually discriminated against the Cronbach’s α coefficient, where when the coefficient is larger than 0.7, it satisfied the reliability verification. When the value of the construct reliability (*CR*) is more than 0.8 and the average variance extracted (*AVE*) is larger than 0.5, it can be tested whether the data is suitable for factor analysis, thus determining whether the data meet the validity criteria. The calculation formula of Cronbach’s α is shown in Formula (6).

(6) $$\alpha =(1-\frac{\sum S_{n}^{2}}{S_{T}^{2}})\times \frac{m}{m-1}$$

The number of “*n*” is the total number of indicator factor categories in the study. For example, the maximum value of *n* in this study was 27. $S_{n}^{2}$ represents the variance of the score at the nth indicator, and $S_{T}^{2}$ represents the total score of all indicators.

The results of the reliability and validity analysis of data from this study are shown in Table 2.

#### 5.1.2. Validity Test Result Analysis

After obtaining a batch of data, it is necessary to verify the validity. According to previous studies, validity can be divided into three categories: (i) validity structure, (ii) content validity, and (iii) criterion relevance.

High validity of the measurement tools implies a high sensitivity of the indicator coefficients between the quantitative measures and their corresponding dimension. This study analyzed the correlation between influencing factor indicators and corresponding data dimensions with Pearson’s product-moment correlation coefficient, where Pearson’s product-moment correlation coefficient test is verified by calculating the value of *t*. The calculation are done using Formulas (7) and (8).

(7) $$P_{r}=\left(\sum_{n=1}^{m}\left(a_{n}-\bar{a}\right)\left(b_{n}-\bar{b}\right)\right){\left(\sqrt{\sum_{n=1}^{m}{\left(a_{n}-\bar{a}\right)}^{2}\sum_{n=1}^{m}{\left(b_{n}-\bar{b}\right)}^{2}}\right)}^{-1}$$

(8) $$t={\left(\sqrt{1-P_{r}^{2}}\right)}^{-1}\left(P_{r}\sqrt{m-2}\right)$$

In the above formulas, m represents the total number of indicator factors. The values of $P_{r}$ are in the range of [0, 1]. Indicator factors are ranked according to the value of $P_{r}$, where the largest means the most significant correlation of the indicator factor. The results of the validity test for the data are shown in Table 3.

The validity test of the observed variable is the factor that constructs the AVE values of latent variables of different dimensions and is a measure of convergent validity; therefore, the validity test value of the observed variable should be consistent with the AVE, which should be greater than 0.5. Since the validity test value of HA5 was 0.495, which is less than 0.01 from the standard range of values, and the AVE of the corresponding dimension was greater than 0.5, the observed variable was considered to have convergent validity within the standard range due to the small error [48].

From the analysis results in the table, it can be obtained that the questionnaire data was reliable and valid for solving the structural equation model of this study.

### 5.2. SEM Solution of Factors Causing Unsafe Behavior of Prefabricated Building Components Hoisting

#### 5.2.1. Model Verification

In this study, a correlation uniqueness model was established based on the logical relationship of the evaluation system. However, the rationality of the CTCM model was difficult to verify. Laura Castro-Schilo [54] used the T-rule test method to solve the value estimation of a CTCM model in their study in 2013, where *t* represented the number of free parameters. The calculation formula for T-rule is Formula (9).

(9) t≤p(p+1)+p

In the evaluation index system of this study, there were five first-level indicators, and a total of 27 second-level indicators were derived. The model data showed that there were 32 exogenous variables and 8 latent variables, the number of which is represented by p; there were 31 endogenous variables, whose number is represented by q. The number of data points *Q* is calculated using Formula (10).

(10) Q=0.5(p+q+1)(p+q)

The *t*-rule has the following provisions for values: (1) When *t* > *Q*, it means that the model is insufficiently recognized and unreliable. (2) When *t* ≤ *Q*, the model is over-identified and can be further analyzed and verified. According to the preliminary calculation, the number of model parameters is shown in Table 4.

In this study, there were 36 parameters with a path coefficient of 1, and the synthesis of free parameters was 36, of which, the number of variances was 32, the number of free paths was 32, and the covariance was 4. Therefore, the total number of free parameters of this model was 108, of which 68 parameter values needed to be estimated. It was calculated that *Q* = 0.5 × (8 + 31 + 1) × (8 + 31) = 496 and *t* = 68. *t* was much smaller than *Q*, which is in line with the previous research; the next calculation and analysis could, therefore, be carried out.

#### 5.2.2. Selection of Model Evaluation Criteria

The reasonableness of a model is indicated by the fitting degree between the analyzed data and the model, which mainly includes the relative fit and the absolute fit. In the absolute fit, the indicators that are often used in data analysis are as follows.

Chi-square $x^{2}$: When the chi-square value is small, the actual data and the hypothetical models in the research are quite consistent; *DF* stands for degrees of freedom, and CMIN also refers to the chi-square value. When the ratio of the chi-square to the degrees of freedom satisfies 1<CMIN/DF<2, the fitting degree of the model is optimal, and the estimated root mean square (*RMSEA*) obtained in this state has the best measure of the fitting. The calculation of *RMSEA* is done using Formula (11), where n represents the number of samples.

(11) $$RMSEA=\sqrt{max\left(\frac{x^{2}-DF}{DF\left(n-1\right)},0\right)}$$

Comparative fitting index (CFI), incremental fitting index (IFI), and normal fitting index (NFI) are indicators of relative fit. According to previous studies, IFI and CFI are constrained below 1. When both indexes are larger than 1, the model should be corrected to avoid excessive bias. NFI is calculated from the chi-square value of the total parameters of the sample, which represents the difference in the chi-square value between the standard model and the research hypothesis model. $x_{I}^{2}$ is the chi-square value of the standard model, and $x_{N}^{2}$ is the chi-square value of the hypothetical model in the study. The calculation of NFI is done using Formula (12).

(12) $$NFI=\frac{\chi_{N}^{2}-\chi_{I}^{2}}{\chi_{N}^{2}}$$

This research calculated the sample data with AMOS 26. The model adaptation index selection and acceptable range are shown in Table 5.

#### 5.2.3. SEM Evaluation of Unsafe Behaviors of Prefabricated Building Components Hoisting

For the model used in this study, the internal structure should be fitted. The importance of each index and potential variables and the significance of the model parameter data can be represented by the fit of the internal structure. The critical value (C.R.) represents the significance of the index factors that can be calculated from $C.R.=\mathrm{Estimate}{\left(S.E.\right)}^{-1}$, in which S.E. represents the standard deviation. It is necessary to calculate the variance and standard deviation of the initial model, and when there is a negative standard deviation and a standard regression coefficient greater than 1 (generally greater than 0.95), the model needs to be corrected before evaluation. The SEM analysis of the variance caused by the prefabricated building hoisting accidents is shown in Table 6.

When inputting the original data into AMOS 26, if there were a violation of the estimation, the internal variables and estimation errors needed to be adjusted. According to the table, it was appropriate to establish association relationships between $e_{3}$ and $e_{4}$, $e_{9}$ and $e_{10}$, $e_{20}$ and $e_{21}$, and path adjustments. The path significance of the modified model was verified. Table 7 shows the path and significance test of the modified model caused by the prefabricated building hoisting accidents. Table 8 shows the standardized estimation of the modified model of the cause of the prefabricated building hoisting accidents.

**Figure 7:**
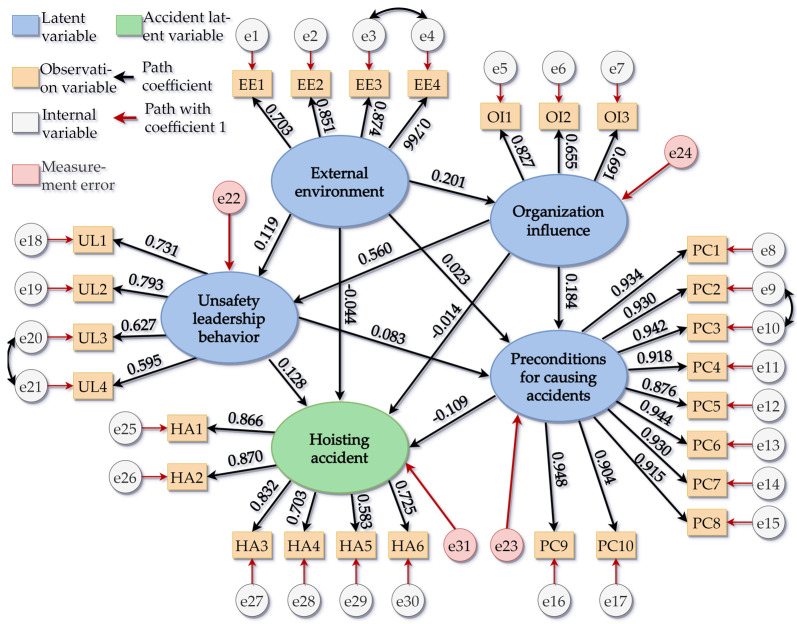
The modified SEM of the prefabricated building hoisting accidents.

As can be seen from the above table, the error variance values of the modified model were not negative, with a range between 0.034 and 0.164. Furthermore, the values of the standardized valuation range between −0.109 and 0.948, which is consistent with the standardized valuation not being greater than 0.95. The modified SEM of the prefabricated building hoisting accidents is shown in Figure 7.

### 5.3. Results Analysis

This study verified and analyzed factors such as the external environment, organization, unsafe leadership behaviors, prerequisites of the accidents, and the accident itself in the prefabricated building hoisting accidents system. The numerical values of the direct effect index, the indirect effect index, and the total effect index of the fitting results are shown in Table 9.

The data in the above table represent the conduction path influence coefficients; the larger the value, the stronger the interference of the factor to the conduction path, and the more obvious its correlation. When problems occur in the upstream factors of the conduction path in the actual construction project, it is highly likely to affect the factors downstream of the conduction path to produce safety hazards, thus leading to safety accidents [46]. The larger the influence coefficient of the conduction path, the greater the possibility of the associated influence. The magnitude of the value is relative and will vary with the number of influencing factors and evaluation systems in different models.

From the data in the above table, we can see that the direct influence path coefficients of the external environment on unsafe leadership behaviors, accident prerequisites, and hoisting accidents were 0.113, 0.056, and 0.018, among which, the coefficient of external environment was the largest, and thus it can be considered that the external environment had a great influence on unsafe leadership behaviors, while the direct influence coefficient of external environment on the organizational factors was 0, and thus it can be considered that changes in the external environmental factors did not interfere with the organizational factors. In terms of indirect influence, the external environment had a great influence on the organizational factors, where the value was 0.183. Moreover, unsafe leadership behavior was the biggest safety damage point for the organizational factors, where the value was 0.098. Regarding the total impact effect, the influences of the external environment, organizational factors, prerequisites of accidents, and unsafe leadership behaviors on hoisting accidents were arranged as such in descending order.

Through the analysis of the prefabricated building components hoisting accidents SEM, it was found that the impact paths of accidents were EE-OI-PC-HA, EE-UL-HA, EE-HA, EE-PC-HA, OI-PC-HA, OI-HA, OI-UL-HA, UL-HA, UL-PC-HA, and PC-HA, among which, the numbers of paths that included the external environment, organizational factors, unsafe leadership behaviors, and prerequisite of accidents were 4, 3, 2, and 1, respectively. Therefore, the analysis of SEM of prefabricated building components hoisting accidents highlighted the correctness of the HFACS-PH and emphasized that construction units need to pay attention to the five stages proposed in this study to ensure the health and safety of construction workers.

## 6. Discussion

### 6.1. Prevention and Control System for Hoisting Accidents of Prefabricated Building Components

From the SEM analysis results of the above-mentioned prefabricated-building-component-hoisting accidents, it can be seen that the bottom-level influencing factor in all conduction paths was HA. From Figure 7, there was an irreversible conduction path between OI and UL; therefore, the longest path in the SEM of the causes of hoisting accidents of prefabricated building components was EE-OI-UL-PC-HA, among which, the number of prerequisite factors of EE, OI, UL, PC, and HA were 0, 1, 2, 3, and 4. It can be seen from Table 9 that each risk cause had different levels of influence on its lower-level factors, which are the factors that construction companies should investigate first when controlling and preventing occupational hazards caused by hoisting safety accidents. For example, when there is a large change in EE, UL should be prevented and controlled during the remediation process of EE.

**Figure 8:**
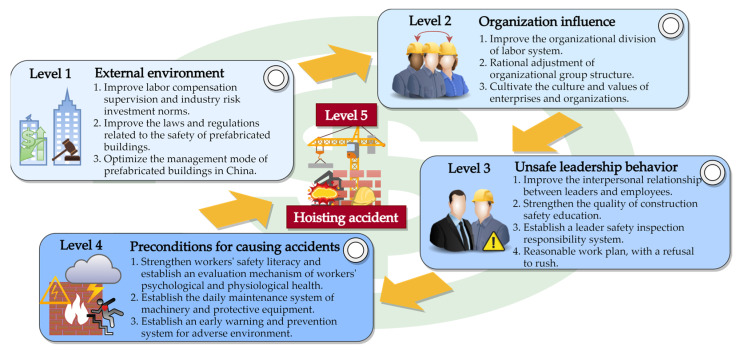
Prefabricated-building-component-hoisting accidents prevention and control system.

In summary, by analyzing the HFACS-PH and SEM of the causes of prefabricated building hoisting accidents, this research concluded that the four preconditions for hoisting accidents were unsafe leadership behaviors, organizational factors, prerequisites for the accidents, and the external environment. Combining these four conditions, a prefabricated-building-component-hoisting accidents prevention and control system was established. The system model is shown in Figure 8.

### 6.2. Influence of External Environmental Factors

(1) Economic factors

In addition to improving financial contract regulations, financial auditing must also be strengthened to ensure the quality and quantity of materials and machinery. In terms of salary, it should be paid on time to reduce the psychological pressure of workers and avoid them from presenting extreme behaviors during the work process that may lead to engineering safety accidents (Wang, H., 2018) [58].

(2) Policy factors

The government should choose a suitable incentive policy and create the applicable laws and regulations related to the safety of prefabricated building construction to regulate the construction safety system [50]. In addition, it should also make strategies according to the relationship of mutual influence between the interests of all parties under the influence of incentive policies proposed by previous scholars to improve the efficiency of safety supervision and reduce the risk of crane accidents as much as possible.

(3) Industry management and historical factors

The management mode of prefabricated construction projects needs to be optimized. For example, the most popular prefabricated projects models, namely, EPC and BOT, can be innovated, and hoisting construction methods and process planning can also be improved. The innovation of industry technology and management gets rid of the traditional frame relying on experts’ experience to make decisions, making the development of prefabricated construction projects safer, more economical, and sustainable, which is of great significance to the development of long-term social benefits.

### 6.3. Influence of Organizational Factors

(1) Perfect organization and division of the labor system

In the prefabricated construction system, a complete division of labor is required to avoid overlapping responsibilities. For example, the financial duties and material procurement duties should be firmly eliminated by the same person to avoid the selection of inferior materials and mechanical products due to personal interests, which may lead to hoisting accidents.

(2) Rationalization of organization structure

Reasonable allocation of organizational management can also reduce hoisting accidents. In the horizontal and vertical organizational structure, the responsibilities of each process should be clearly defined (Cheung, C.M., 2021) [59]. With clear responsibilities, managers will strictly require relevant rules and regulations to standardize the whole construction, thus reducing the probability of accidents.

(3) Foster representative organizational values

Excellent organizational values can discipline workers’ behaviors and make them strictly adhere to industry standards. Most accidents are caused by human factors, and a good organizational culture can promote the development process of workers and the sustainable benefits of prefabricated construction. The most important aspect that distinguishes the prefabricated construction model from the traditional one is the need for highly skilled workers; therefore, sound organizational culture values can enhance the cohesiveness of working teams, reducing unsafe behaviors (Huang, Y.H., 2021) [60].

### 6.4. Influence of Unsafe Leadership Behaviors

Each of Maslow’s five principles of needs is related to professional leadership behaviors, which are analyzed in the following four points.

(1) Improving the relationship between leaders and workers

In professional work, leaders often determine the quality of workers’ high-level needs, among which, being respected and gaining personal value are important. If workers can not get the respect they require from their leaders, they will be depressed, affecting work efficiency, or even worse, they will develop depressive tendencies, leading to extreme behaviors, which can result in hoisting accidents and have a bad impact on the occupational safety of all staff. The relationship between leaders and workers can also be developed into the social needs of workers. Satisfying the social demands of workers and expanding the professional formal groups into informal friendship groups will promote the sense of responsibility of workers and make workers pay more attention to avoid hoisting accidents (Lingard, H., 2017) [61].

(2) Strengthen safety education

Leaders should actively advocate occupational safety education. In the process of education, workers’ mental states will likewise be changed by the leaders’ recognition and the satisfaction of gaining knowledge. With the realization of self-worth, workers feel that the industry they engaged in has a great significance of social contribution, which will greatly enhance the enthusiasm and self-confidence of their work and make workers pay more attention to occupational safety and avoid hoisting accidents.

(3) Strengthening safety inspections

To meet the safety needs of employees, leaders should develop safety inspection systems and strictly implement them. A safe working environment will encourage workers to have a stable mentality and enable them to focus more on their work.

(4) Make a reasonable work plan

Leaders should develop a reasonable work plan to meet workers’ physiological needs. Unreasonable work plans will have a bad impact on workers’ diet, work, rest, and mental state, which will probably lead to hoisting accidents.

### 6.5. Influence of Prerequisites

(1) The human factor of the preconditions for accidents

Regarding the human factors in the prerequisites for accidents in prefabricated construction projects, we should strengthen the personal safety literacy of workers to improve their physical and mental quality and their ability to resist frustration. Furthermore, a healthy competitive relationship should be established to strengthen the morality of workers and avoid some illegal behaviors and malicious competition (He, Q.H., 2016) [62], thus leading to unsafe behaviors. A complete and sound incentive mechanism should be established based on the current safe and civilized construction bonus system to quantify the performance of workers, and the bonus should be paid according to the ratio of their safety duties (Barling, J., 2002) [51]. It is also necessary to establish a psychological guidance and consultation system to monitor the mental health of workers to develop their ability to respond to safety accidents, improving the efficiency of accident emergency responses. The construction unit shall conduct annual physical examinations for workers and increase the physical health monitoring in the daily management so as to control the physical health of workers in a timely manner to avoid the possibility of hoisting safety accidents due to the concealment of physiological diseases. A psychological cognitive evaluation system for workers should also be established during the periodic training to assess the psychological condition of workers. The construction company should also establish mental health management departments with an occupational physician as an official job, and develop proper mental health management systems and physiological abnormality files for workers to avoid them engaging in work beyond their capabilities so as to reduce workers’ occupational safety hazards.

(2) The equipment factors of the preconditions for accidents

For the equipment factors in the prerequisites of accidents in the prefabricated building project, the construction unit shall establish a system for the regular maintenance of machinery, materials, and tools to prevent safety accidents during the hoisting process. Equipment with defects or minor failures should be repaired in time (Cheung, C.M., 2020) [53], and equipment with aging or structural damage should be replaced to avoid serious safety accidents due to engineering costs savings.

(3) Environmental factors of accident preconditions

For the environmental factors, first, a weather detection and warning system should be established (Cheung, C.M.,2020) [53]. Taking the intra-city tornado in Shenyang due to strong convective weather into consideration, an extreme weather emergency response system should be established to guarantee the safety of the hoisting process. Second, the tower crane location should be rationally decided and new algorithms should be used to calculate the working path of the tower crane and the spherical working area to avoid being blocked. The storage point of components should be cleaned and monitored daily to avoid falling accidents during the hoisting process due to the quality problems caused by improper storage.

## 7. Conclusions

Based on the research of HOFs, this study established the HFACS-PH model of unsafe behaviors and related influencing factors for hoisting prefabricated components (PCs) of prefabricated buildings by investigating and analyzing the construction engineering accidents in China over the past 11 years. The authors also established an evaluation system of accident factors for the hoisting of prefabricated building components and designed questionnaires to consult experts and practitioners to verify the validity of the HFACS-PH model.

According to the survey data from the experts, it was found that there were 10 paths among the external environment, organizational factors, unsafe leadership behaviors, and the prerequisite of accidents that could lead to hoisting safety accidents that seriously threatened the safety of workers. Among them, both external factors and organizational factors can result in accidents through unsafe leadership behaviors and prerequisites. According to the path coefficients, the unsafe leadership behaviors and the prerequisites of accidents were the most significant influence paths. According to the results of the SEM analysis and significance index, the model was revised and a qualitative and quantitative model of the causes of lifting accidents was obtained. By modifying the model with the results of SEM analysis and significance indicators, the qualitative and quantitative models of the cause of the hoisting accidents were obtained.

Based on the HFACS-PH model and the qualitative and quantitative causative models established in this study, the prevention and control system of PC hoisting in prefabricated buildings was constructed. The prevention and control system was established according to the numerical magnitude of the standardized regression coefficient regarding establishing regulations, supervision, improving management systems, increasing economic investment in safety, cultivating workers’ occupational safety awareness, and rational planning of construction arrangements, which adds a theoretically significant guarantee for the safety of workers in subsequent prefabricated construction projects. In addition, the construction unit can formulate the safety code of conduct for workers in the hoisting construction phase based on the HFACS-PH model and use it as the theoretical basis for the formulation and analysis of the accident reports.

The follow-up engineering accident reports and statistical data collected according to the framework of this study can be used as the data basis for the knowledge map of the cause of prefabricated building hoisting accidents.

## Figures and Tables

**Table 1:** Factors for accidents of prefabricated building components hoisting.

Latent Variable	Variable Symbol	Observed Variable	Variable Symbol	Label	Point Range
External environment	EE	Economic factors	EE1	e1	One point to five points
		Policy factors	EE2	e2	
		Industry management	EE3	e3	
		Historical factors	EE4	e4	
Organization influence	OI	Management process	OI1	e5	
		Organizational climate	OI2	e6	
		Resource management	OI3	e7	
Unsafe leadership behaviors	UL	Inadequate supervision	UL1	e8	
		Improper plan	UL2	e9	
		Failure to correct problems	UL3	e10	
		Violation of supervision	UL4	e11	
Preconditions for causing accidents	PC	Professional level of workers	PC1	e12	
		Mental state of workers	PC2	e13	
		Physiological state of workers	PC3	e14	
		Component production quality	PC4	e15	
		Strength of the connection point	PC5	e16	
		Hoisting equipment status	PC6	e17	
		Safety protection measures	PC7	e18	
		Operating conditions	PC8	e19	
		Rationality of the layout	PC9	e20	
		Component storage environment	PC10	e21	
Hoisting accidents	HA	Falling from a height	HA1	e22	
		Connection failure	HA2	e23	
		Pinch	HA3	e24	
		Mechanical failure	HA4	e25	
		Hoisting load dumping	HA5	e26	
		Hoisting load falls off	HA6	e27	

**Table 2:** Results of the reliability and validity analysis of the questionnaire data.

Dimension	Cronbach’s *α*	CR	AVE
External environment	0.876	0.9125	0.7229
Organization influence	0.780	0.8446	0.6446
Unsafe leadership behaviors	0.786	0.8437	0.5748
Preconditions for causing accidents	0.985	0.986	0.8756
Hoisting accidents	0.895	0.9188	0.655
All indexes	0.877	-	-

Note: “-” stands for null.

**Table 3:** Questionnaire data validity test result.

Observed Variable	Variable Symbol	Validity Test Value	Observed Variable	Variable Symbol	Validity Test Value
Economic factors	EE1	0.670	Component production quality	PC4	0.861
Policy factors	EE2	0.768	Strength of the connection point	PC5	0.804
Industry management	EE3	0.794	Hoisting equipment status	PC6	0.895
Historical factors	EE4	0.721	Safety protection measures	PC7	0.882
Management process	OI1	0.738	Operating conditions	PC8	0.863
Organizational climate	OI2	0.727	Rationality of the layout	PC9	0.924
Resource management	OI3	0.687	Component storage environment	PC10	0.845
Inadequate supervision	UL1	0.665	Falling from a height	HA1	0.761
Improper plan	UL2	0.693	Connection failure	HA2	0.761
Failure to correct problems	UL3	0.620	Pinch	HA3	0.737
Violation of supervision	UL4	0.563	Mechanical failure	HA4	0.597
Professional level of workers	PC1	0.912	Hoisting load dumping	HA5	0.495
Mental state of workers	PC2	0.942	Hoisting load falls off	HA6	0.655
Physiological state of workers	PC3	0.930			

**Table 4:** Model parameter number.

	Weights	Covariances	Variances	Total
Fixed	36	0	0	36
Unfixed	32	4	32	68
Total	68	4	32	104

**Table 5:** Selection and acceptable range of the model adaptation index.

Index Name	Acceptable Range	Supporting Literature	Fit Value
*x*^2^/df	≤3.00 good fit	[55,56,57]	2.131
GFI (goodness of fit)	>0.80 good fit	[55,56,57]	0.849
AGFI (adjusted goodness of fit)	>0.80 good fit	[55,57]	0.818
IFI (incremental fit index)	>0.90 good fit	[55,57]	0.949
TLI (Tucker–Lewis index)	>0.90 good fit	[55,57]	0.942
CFI (comparative fit index)	>0.90 good fit	[55,57]	0.948
RMSEA (root-mean-square error approximation)	<0.05 good fit	[55,57]	0.065
	<0.08 fair fit		
	<0.10 normal fit		
RMR (standardized root-mean-square residual)	<0.05 good fit	[55,56,57]	0.047
	<0.08 fair fit		

**Table 6:** SEM variance analysis of the causes of hoisting accidents in prefabricated buildings.

Label	Estimate	S.E.	C.R.	*p*	Label	Estimate	S.E.	C.R.	*p*
EE	0.586	0.077	7.658	***	e16	0.105	0.011	9.908	***
e1	0.387	0.038	10.098	***	e17	0.232	0.021	10.895	***
e2	0.306	0.040	7.602	***	e18	0.492	0.063	7.827	***
e3	0.239	0.036	6.708	***	e19	0.426	0.077	5.519	***
e4	0.477	0.051	9.389	***	e20	0.540	0.058	9.382	***
e5	0.330	0.092	3.584	***	e21	0.799	0.085	9.411	***
e6	0.586	0.077	7.658	***	e22	0.365	0.080	4.580	***
e7	0.617	0.093	6.618	***	e23	0.970	0.102	9.474	***
e8	0.119	0.012	9.985	***	e24	0.542	0.114	4.755	***
e9	0.069	0.008	9.125	***	e25	0.351	0.042	8.266	***
e10	0.080	0.008	9.680	***	e26	0.271	0.033	8.150	***
e11	0.163	0.015	10.789	***	e27	0.396	0.044	9.087	***
e12	0.269	0.024	11.088	***	e28	0.532	0.051	10.490	***
e13	0.119	0.012	10.339	***	e29	0.612	0.056	10.973	***
e14	0.164	0.015	10.579	***	e30	0.582	0.057	10.233	***
e15	0.204	0.019	10.795	***	e31	1.030	0.119	8.653	***

Note: “***” indicates that the probability of getting a critical ratio as large as the C.R. value as an absolute value was less than 0.001.

**Table 7:** Modified model path and significance test of the causes of prefabricated building hoisting accidents.

Routing	Estimate	S.E.	C.R.	*p*	Routing	Estimate	S.E.	C.R.	*p*
OI←EE	0.183	0.067	2.729	0.006	PC3←PC	0.983	0.034	29.331	***
UL←EE	0.109	0.062	1.743	0.081	PC4←PC	0.920	0.036	25.278	***
UL←OI	0.561	0.110	5.097	***	PC5←PC	0.926	0.042	22.302	***
PC←UL	0.112	0.125	0.892	0.373	PC6←PC	0.972	0.035	27.550	***
PC←OI	0.249	0.125	1.991	0.047	PC7←PC	1.006	0.038	26.263	***
PC←EE	0.028	0.082	0.345	0.730	PC8←PC	1.006	0.040	25.122	***
HA←OI	−0.019	0.134	−0.139	0.890	PC9←PC	1.065	0.037	28.953	***
HA←PC	−0.110	0.067	−1.644	0.100	PC10←PC	1.000			
HA←UL	0.175	0.134	1.311	0.190	UL1←UL	1.000			
HA←EE	−0.055	0.087	−0.626	0.531	UL2←UL	1.131	0.122	9.280	***
EE1←EE	0.746	0.064	11.652	***	UL3←UL	0.785	0.086	9.090	***
EE2←EE	1.089	0.077	14.112	***	UL4←UL	0.879	0.101	8.698	***
EE3←EE	1.066	0.075	14.314	***	HA1←HA	1.000			
EE4←EE	1.000				HA2←HA	0.893	0.048	18.724	***
OI1←OI	1.124	0.164	6.857	***	HA3←HA	0.918	0.054	17.016	***
OI2←OI	0.884	0.095	9.331	***	HA4←HA	0.702	0.053	13.134	***
OI3←OI	1.000				HA5←HA	0.547	0.054	10.185	***
PC1←PC	1.084	0.038	28.543	***	HA6←HA	0.782	0.058	13.604	***
PC2←PC	1.038	0.034	30.248	***					

Note: “***” indicates that the probability of getting a critical ratio as large as the C.R. value as an absolute value was less than 0.001.

**Table 8:** Standardized estimates of modified models caused by hoisting accidents in prefabricated buildings.

Routing	Estimate (Standardized)	Routing	Estimate (Standardized)
OI←EE	0.201	PC3←PC	0.942
UL←EE	0.119	PC4←PC	0.918
UL←OI	0.560	PC5←PC	0.876
PC←UL	0.083	PC6←PC	0.944
PC←OI	0.184	PC7←PC	0.930
PC←EE	0.023	PC8←PC	0.915
HA←OI	−0.014	PC9←PC	0.948
HA←PC	−0.109	PC10←PC	0.904
HA←UL	0.128	UL1←UL	0.731
HA←EE	−0.044	UL2←UL	0.793
EE1←EE	0.703	UL3←UL	0.627
EE2←EE	0.851	UL4←UL	0.595
EE3←EE	0.874	HA1←HA	0.866
EE4←EE	0.766	HA2←HA	0.870
OI1←OI	0.827	HA3←HA	0.832
OI2←OI	0.655	HA4←HA	0.703
OI3←OI	0.691	HA5←HA	0.583
PC1←PC	0.934	HA6←HA	0.725
PC2←PC	0.930		

**Table 9:** Direct influence effect, indirect influence effect, and total impact effect numerical table.

Standardized Direct Influence Effect Values					
	EE	OI	UL	PC	HA
OI	0	0	0	0	0
UL	0.113	0	0	0	0
PC	0.056	0.046	0	0	0
HA	0.018	0.045	0.009	0	0
**Standardized Indirect Influence Effect Values**					
	**EE**	**OI**	**UL**	**PC**	**HA**
OI	0.183	0	0	0	0
UL	0.098	0.561	0	0	0
PC	0.041	0.266	0.112	0	0
HA	0.014	0.002	0.154	0.110	0
**Total Impact Effect Values**					
	**EE**	**OI**	**UL**	**PC**	**HA**
OI	0.183	0	0	0	0
UL	0.211	0.561	0	0	0
PC	0.097	0.312	0.112	0	0
HA	0.032	0.047	0.163	0.110	0

## Data Availability

The case analysis data used to support the findings of this study are available from the corresponding author upon request.

## References

[1] Zuo J., Pullen S., Rameezdeen R., Bennetts H., Wang Y., Mao G., Zhou Z., Du H., Duan H. (2017). Green building evaluation from a life-cycle perspective in Australia: A critical review. Renew. Sustain. Energy Rev..

[2] Shi Q., Chen J., Shen L. (2017). Driving factors of the changes in the carbon emissions in the Chinese construction industry. J. Clean. Prod..

[3] Chen L.Y., Gao X., Hua C.X., Gong S.T., Yue A.B. (2021). Evolutionary process of promoting green building technologies adoption in China: A perspective of government. J. Clean. Prod..

[4] Ministry of Housing and Urban-Rural Development of the People’s Republic of China. http://www.mohurd.gov.cn/.

[5] 2020 National Construction Safety Accident Data “Released”, It Is Imperative to Promote VR Site Safety Training. http://www.ycc333.com/Article/2020qgjzaq.html.

[6] Wang Z.H., Li L., Zhang Y.X., Wang W.T. (2019). Bond-slip model considering freeze-thaw damage effect of concrete and its application. Eng. Struct..

[7] Wang J., Liao P.-C. (2021). Re-Thinking the Mediating Role of Emotional Valence and Arousal between Personal Factors and Occupational Safety Attention Levels. Int. J. Environ. Res. Public Health.

[8] Tam V.W.Y., Tam C.M., Zeng S.X., Ng W.C.Y. (2007). Towards adoption of prefabrication in construction. Build. Environ..

[9] Hong J.K., Shen G.Q.P., Li Z.D., Zhang B.Y., Zhang W.Q. (2018). Barriers to promoting prefabricated construction in China: A cost-benefit analysis. J. Clean. Prod..

[10] Wang X.W., Sun Y.C., Liu Y. (2020). Research on Influencing Factors of Unsafe Behavior of Prefabricated Building. IOP Conference Series: Earth and Environmental Science.

[11] Li H., Lu M.J., Hsu S.C., Gray M., Huang T. (2015). Proactive behavior-based safety management for construction safety improvement. Saf. Sci..

[12] Haslam R., Hide S., Gibb A., Gyi D., Pavitt T., Atkinson S., Duff A.R. (2005). Contributing factors in construction accidents. Appl. Ergon..

[13] Harvey E., Waterson P., Dainty A. (2019). Impact of the ‘Contributing Factors in Construction Accidents’ (ConCA) Model. Adv. Intell. Syst..

[14] Goncalves A.P.G., Waterson P., Jun G.T. (2021). Improving accident analysis in construction—Development of a contributing factor classification framework and evaluation of its validity and reliability. Saf. Sci..

[15] Soltanzadeh A., Mohammadfam I., Moghimbeigi A., Akbarzadeh M., Ghiasvand R. (2016). Key factors contributing to accident severity rate in construction industry in Iran: A regression modelling approach. Arh. Hig. Rada Toksikol..

[16] Suraji A., Duff A.R., Peckitt S.J. (2001). Development of causal model of construction accident causation. J. Constr. Eng. Manag..

[17] Suo Q.H., Zhang D.M. (2017). Investigation and identification of factors affecting migrating peasant workers’ usage of safety footwear in the Chinese construction industry. Int. J. Occup. Saf. Ergon..

[18] Wu W.W., Yang H.J., Chew D.A.S., Yang S.H., Gibb A.G.F., Li Q.M. (2010). Towards an autonomous real-time tracking system of near-miss accidents on construction sites. Autom. Constr..

[19] Mohamed S. (2002). Safety climate in construction site environments. J. Constr. Eng. Manag..

[20] Yi W., Chan A.P.C. (2013). Optimizing work-rest schedule for construction rebar workers in hot and humid environment. Build. Environ..

[21] Lu Y., Zhu Y.Q. (2021). Integrating Hoisting Efficiency into Construction Site Layout Plan Model for Prefabricated Construction. J. Constr. Eng. Manag..

[22] Adrian A., Utamima A., Wang K.J. (2015). A comparative study of GA, PSO and ACO for solving construction site layout optimization. KSCE J. Civ. Eng..

[23] Al Hawarneh A., Bendak S., Ghanim F. (2019). Dynamic facilities planning model for large scale construction projects. Autom. Constr..

[24] Cheng B., Lu K., Li J., Chen H., Luo X., Shafique M. (2022). Comprehensive assessment of embodied environmental impacts of buildings using normalized environmental impact factors. J. Clean. Prod..

[25] Huang C., Wong C.K. (2015). Optimisation of site layout planning for multiple construction stages with safety considerations and requirements. Autom. Constr..

[26] Huang C., Wong C.K., Tam C.M. (2011). Optimization of tower crane and material supply locations in a high-rise building site by mixed-integer linear programming. Autom. Constr..

[27] Liu Z.S., Meng X.T., Xing Z.Z., Jiang A.T. (2021). Digital Twin-Based Safety Risk Coupling of Prefabricated Building Hoisting. Sensors.

[28] Zhao Y.H., Cao C.F., Liu Z.S., Mu E.Y. (2021). Intelligent Control Method of Hoisting Prefabricated Components Based on Internet-of-Things. Sensors.

[29] Arashpour M., Wakefield R., Blismas N., Minas J. (2015). Optimization of process integration and multi-skilled resource utilization in off-site construction. Autom. Constr..

[30] Ma Z.L., Cai S.Y., Mao N., Yang Q.L., Feng J.G., Wang P.Y. (2018). Construction quality management based on a collaborative system using BIM and indoor positioning. Autom. Constr..

[31] Shin I.J. (2015). Factors that affect safety of tower crane installation/dismantling in construction industry. Saf. Sci..

[32] Chi S., Han S., Kim D.Y. (2013). Relationship between Unsafe Working Conditions and Workers’ Behavior and Impact of Working Conditions on Injury Severity in US Construction Industry. J. Constr. Eng. Manag..

[33] Xia N.N., Zou P.X.W., Liu X., Wang X.Q., Zhu R.H. (2018). A hybrid BN-HFACS model for predicting safety performance in construction projects. Saf. Sci..

[34] Xu R.H., Luo F., Chen G.M., Zhou F.H., Abdulahi E.W. (2021). Application of HFACS and grounded theory for identifying risk factors of air traffic controllers’ unsafe acts. Int. J. Ind. Ergon..

[35] Hsieh M.-C., Wang E.M.-Y., Lee W.-C., Li L.-W., Hsieh C.-Y., Tsai W., Wang C.-P., Huang J.-L., Liu T.-C. (2018). Application of HFACS, fuzzy TOPSIS, and AHP for identifying important human error factors in emergency departments in Taiwan. Int. J. Ind. Ergon..

[36] Liu R.L., Cheng W.M., Yu Y.B., Xu Q.F. (2018). Human factors analysis of major coal mine accidents in China based on the HFACS-CM model and AHP method. Int. J. Ind. Ergon..

[37] Lenne M.G., Salmon P.M., Liu C.C., Trotter M. (2012). A systems approach to accident causation in mining: An application of the HFACS method. Accid. Anal. Prev..

[38] Xie Q.H., Xia N.N., Yang G.S. (2022). Do Family Affairs Matter? Work-Family Conflict and Safety Behavior of Construction Workers. J. Manag. Eng..

[39] Liang H.K., Shi X.X., Yang D.H., Liu K.N. (2022). Impact of mindfulness on construction workers’ safety performance: The mediating roles of psychological contract and coping behaviors. Saf. Sci..

[40] Fugas C.S., Silva S.A., Melia J.L. (2012). Another look at safety climate and safety behavior: Deepening the cognitive and social mediator mechanisms. Accid. Anal. Prev..

[41] Gao R., Chan A.P.C., Utama W.P., Zahoor H. (2016). Multilevel Safety Climate and Safety Performance in the Construction Industry: Development and Validation of a Top-Down Mechanism. Int. J. Environ. Res. Public Health.

[42] Guo B.H.W., Yiu T.W., Gonzalez V.A. (2016). Predicting safety behavior in the construction industry: Development and test of an integrative model. Saf. Sci..

[43] Analysis on the Number, Death Toll and Accident Types of Production Safety Accidents in Housing and Municipal Engineering in China. https://www.chyxx.com/industry/202012/915491.html.

[44] National Large-Scale Construction Safety Accidents in 2020-Tower Crane Collapse and High Falling. https://blog.51cto.com/u_14890609/2519824.

[45] Li X.J. (2020). Research on investment risk influence factors of prefabricated building projects. J. Civ. Eng. Manag..

[46] Liu K., Zhao P., Wang H. (2017). Application of SEM based prefabricated concrete structure. J. Civ. Eng. Manag..

[47] Zheng S.Q., Wang D.F., Zuo Q.L., He Q. (2016). Research on Influencing Factors of prefabricated building cost based on SEM. Proj. Manag. Technol..

[48] Song Y., Wang J., Guo F., Lu J., Liu S. (2021). Research on Supplier Selection of Prefabricated Building Elements from the Perspective of Sustainable Development. Sustainability.

[49] General Office of the Ministry of Housing and Urban-Rural Development of the People’s Republic of China (2016). Guiding Opinions of the General Office of the State Council on Vigorously Developing Prefabricated Buildings.

[50] Department of Standards and Quotas, Ministry of Housing and Urban-Rural Development Circular on the Development of Prefabricated Buildings in China in 2020. http://www.mohurd.gov.cn/gongkai/fdzdgknr/tzgg/202103/20210312_249438.html.

[51] Barling J., Loughlin C., Kelloway E.K. (2002). Development and test of a model linking safety-specific transformational leadership and occupational safety. J. Appl. Psychol..

[52] Georgiou K., Nikolaou D.B.T. (2021). Turban. The impact of a training intervention developing psychological capital on job search success. J. Career Dev..

[53] Cheung C.M., Zhang R.P. (2020). How Organizational Support Can Cultivate a Multilevel Safety Climate in the Construction Industry. J. Manag. Eng..

[54] Castro-Schilo L., Grimm K.J., Widaman K.F. (2013). Abstract: Uncrossing the Correlated Trait-Correlated Method Model for Multitrait Multimethod Data. Appl. Ecol. Environ. Res..

[55] Seo H.C., Lee Y.S., Kim J.J., Jee N.Y. (2015). Analyzing safety behaviors of temporary construction workers using structural equation modeling. Saf. Sci..

[56] Al-Mekhlafi A.-B.A., Isha A.S.N., Chileshe N., Abdulrab M., Saeed A.A.H., Kineber A.F. (2021). Modelling the Relationship between the Nature of Work Factors and Driving Performance Mediating by Role of Fatigue. Int. J. Environ. Res. Public Health.

[57] Buniya M.K., Othman I., Durdyev S., Sunindijo R.Y., Ismail S., Kineber A.F. (2021). Safety Program Elements in the Construction Industry: The Case of Iraq. Int. J. Environ. Res. Public Health.

[58] Wang H., Nie W., Cheng W.M., Liu Q., Jin H. (2018). Effects of air volume ratio parameters on air curtain dust suppression in a rock tunnel’s fully-mechanized working face. Adv. Powder Technol..

[59] Cheung C.M., Zhang R.P., Wang R., Hsu S.C., Manu P. (2022). Group-Level Safety Climate in the Construction Industry: Influence of Organizational, Group, and Individual Factors. J. Manag. Eng..

[60] Huang Y.H., Lee J., McFadden A.C., Rineer J., Robertson M.M. (2017). Individual employee’s perceptions of “Group-level Safety Climate” (supervisor referenced) versus “Organization-level Safety Climate” (top management referenced): Associations with safety outcomes for lone workers. Accid. Anal. Prev..

[61] Lingard H., Zhang R.P., Oswald D. (2019). Effect of leadership and communication practices on the safety climate and behaviour of construction workgroups. Eng. Constr. Arch. Manag..

[62] He Q.H., Dong S., Rose T., Li H., Yin Q., Cao D.P. (2016). Systematic impact of institutional pressures on safety climate in the construction industry. Accid. Anal. Prev..

